# Low-cost physiology and behavioral monitor for intravital imaging in small mammals

**DOI:** 10.1117/1.NPh.12.1.015004

**Published:** 2025-01-25

**Authors:** Yuntao Li, Alfredo Cardenas-Rivera, Chang Liu, Zhengyi Lu, Jaime Anton, Mohammed Alfadhel, Mohammad A. Yaseen

**Affiliations:** a Northeastern University, Department of Bioengineering, Boston, Massachusetts, United States; b Massachusetts General Hospital, Harvard Medical School, Athinoula A. Martinos Center for Biomedical Imaging, Charlestown, Massachusetts, United States

**Keywords:** awake mouse imaging, spontaneous motion, pupillometry, optical intrinsic signal, calcium imaging, functional activation

## Abstract

**Significance:**

Functional brain imaging experiments in awake animals require meticulous monitoring of animal behavior to screen for spontaneous behavioral events. Although these events occur naturally, they can alter cell signaling and hemodynamic activity in the brain and confound functional brain imaging measurements.

**Aim:**

We developed a centralized, user-friendly, and stand-alone platform that includes an animal fixation frame, compact peripheral sensors, and a portable data acquisition system. The affordable, integrated platform can benefit imaging experiments by monitoring animal behavior for motion detection and alertness levels as complementary readouts for brain activity measurements.

**Approach:**

A custom acquisition system was designed using a powerful, inexpensive microcomputer. We customized an accelerometer and miniature camera modules for efficient, real-time monitoring of animal motion detection and pupil diameter. We then tested and validated the platform’s performance with optical intrinsic signal imaging and GCaMP fluorescence calcium imaging in functional activation experiments in awake mice.

**Results:**

The integrated platform shows promise for detecting spontaneous motion and pupil dilation while imaging. Stimulus-induced pupil dilation was found to initiate earlier than cortical hemodynamics with a slower rise time. Compared with neuronal calcium response, stimulus-induced pupil dilation initiated later with a slower rise time.

**Conclusions:**

We developed an integrated platform to monitor animal motion and pupil dynamics. The device can be easily coupled and synchronized with optical brain imaging systems to monitor behavior, alertness, and spontaneous motion for awake animal studies.

## 1 Introduction

As the neuroscience community strives to gain more detailed insight into cellular and vascular mechanisms underlying brain function, techniques and practices for measuring brain activity in awake animals have become indispensable.^1^^,^^2^ The confounding effects of anesthesia vary notably with different anesthetics and heterogeneously impact different regions of the brain and over different timescales, and their influence on energy metabolism, hemodynamic signaling, and neuronal circuitry can be especially problematic for investigations of task-evoked neurovascular coupling or microvascular dysfunction.^3^^,^^4^ A wide range of custom head- and body-fixation and habituation protocols have been engineered for imaging studies in awake animals.^5^^,^^6^ Imaging brain activity in awake animals avoids the complications of anesthesia and enables a more accurate correlation between observations of brain pathology with cognitive and behavioral assessments. However, experimental readouts from awake subjects are highly susceptible to perturbations associated with spontaneous, naturally occurring behaviors. Awake animals constantly engage in whisking, grooming, and other spontaneous “fidgeting” motions, and they rapidly fluctuate among different states of alertness. These frequent, transient events can notably alter neuronal and hemodynamic activity or introduce motion artifacts in the experimental readouts,^7^^–^^10^ warranting a large number of experimental trials. This can be particularly problematic for longitudinal studies and investigations exploring task-evoked activation.

Although designing firmer head fixation methods or motion-correction algorithms helps to mitigate their impact, spontaneous fidgeting and variations in alertness notably disrupt optical measurements of brain function and impair robust interpretation of results. These behaviors are naturally unavoidable and warrant the urgent need for robust monitoring methods. In practice, investigators utilize an array of peripheral sensors and cameras to monitor an animal subject’s behavior during imaging experiments. The use of multiple, peripheral sensors can often be cumbersome, particularly if the sensors are bulky or require careful positioning within an optical imaging system’s often-limited sample space. Precise positioning and testing of multiple sensors can prolong experimental setup time. In some labs, each peripheral sensor is recorded with a different acquisition software, often asynchronously from the primary brain imaging measurements, which confounds data processing. Because the duration of the head restraint should be limited to ∼2  h for awake mouse subjects each day,^11^ a lengthy setup and testing process for multiple sensors significantly reduces the available time for viable data acquisition. In addition, the assessment of animal behavior data must usually be performed offline after completing the full imaging experiment. The inability to review measurements immediately after acquisition often results in an insufficient number of valid measurements per experiment, significantly increasing the number of experiments and animal subjects required to complete a study.

Recognition continues to grow for the wide range of factors contributing to fluctuations in brain activity, both under resting conditions and in response to a functional stimulus.^7^^–^^9^ The growing number of physiological and behavioral parameters for monitoring significantly increases the complexity of experimental setups and warrants techniques for centralizing these complementary measurements into a convenient all-in-one system. Zhang et al.^12^ provided detailed guidance to monitor multiple behavioral and physiological metrics concurrently with brain activity measurements using custom data acquisition software, illustrating the indispensable importance of behavioral monitoring and the notable convenience of a robust and centralized acquisition system. In this work, we demonstrate a low-cost platform to streamline multiple readouts of animal behavior from compact peripheral sensors to a single, portable custom acquisition device. The total cost of this platform is under $500, and all data acquisition and processing are performed using a portable microcomputer. The customized, compact sensors can be quickly and easily positioned, greatly reducing imaging preparation time and enabling simple integration with custom and commercial imaging systems. Measurements from our device can be immediately reviewed after completing a sequence of experimental trials, and the recordings can be easily synchronized with readouts from multiple brain imaging modalities such as two-photon microscopy or optical coherence tomography. This work constitutes our pilot efforts to develop a user-friendly, affordable device that easily and efficiently couples with intravital imaging systems to monitor critical physiological and behavioral readouts for awake brain imaging in rodents. To validate its performance, we coupled the device with our custom wide-field optical imaging system (WFOI) for functional activation experiments. Specifically, we used our device to monitor awake mouse behavior while measuring stimulus-induced cortical hemodynamic and neural activity with WFOI in Thy1-GCaMP6f mice. The results confirm that our integrated platform efficiently detects the animal’s spontaneous motion during experiments and the dynamic fluctuations in pupil diameter, demonstrating its convenience and ease of use for physiological monitoring during awake brain imaging experiments.

## 2 Results

**Fig. 1:**
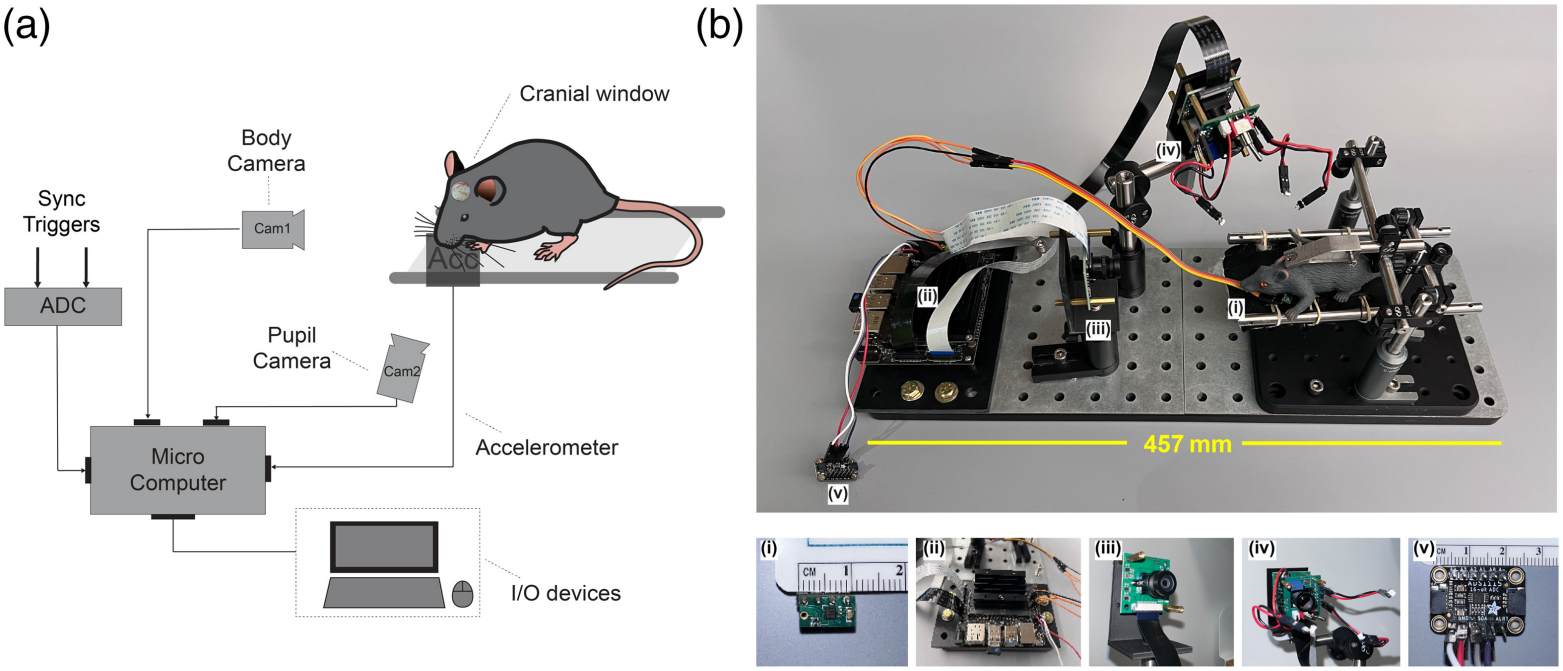
(a) Schematic of the integrated platform setup on an awake mouse with a cranial window. (b) The integrated platform design consists of a data acquisition system and an animal fixation frame. b(i)–b(v) Components of the data acquisition system: (i) BMA250 accelerometer, (ii) Jetson Nano microcomputer, (iii) body camera, (iv) pupil camera, and (v) 16-bit A/D converter. The system’s commercial components are provided in Table S1 in the Supplementary Material.

We designed a user-friendly and low-cost device to monitor animal physiological signals and motion for awake imaging experiments, and we validated its performance with functional stimulation experiments. The general design is shown in Fig. 1(a). It requires minimal setup time and facilitates our neuroimaging measurements by providing real-time feedback on animal behavior.

### 2.1 Hardware Design

#### 2.1.1 Accelerometer

Despite the use of robust head fixation methods, spontaneous movements of the animal’s limbs and body frequently yield motion artifacts in awake brain function measurements, significantly compromising data interpretation and prolonging experimental studies. To detect these movements, we designed a miniature accelerometer board based on the BMA250 sensor (BOSCH, Gerlingen, German). The BMA250 sensor is a compact (2×2×0.1  mm), ultralow power, triaxial accelerometer with high sensitivity (±2  g) and communicates through the digital $I^{2}C$ or SPI interface. We embedded the BMA250 chip on a printed circuit board (10  mm*5  mm), allowing easy placement under the mouse body [Fig. 1(b)i]. To minimize disturbance, we positioned the accelerometer under the animal cradle where the mouse’s forelimbs rest. The BMA250 collects triaxial data at regular intervals. The readings were filtered by BMA250’s internal bandpass filter. The acceleration is calculated by the magnitude of the acceleration vectors in the x-, y-, and z-axes and is presented as the absolute value.

#### 2.1.2 Microcomputer, camera, and LED illumination

To consolidate and synchronize all peripheral sensor measurements into a single data acquisition environment, we utilized a portable and inexpensive microcomputer. Our current design was developed using the Jetson Nano Developer Kit [Fig. 1(b)ii, NVIDIA, Santa Clara, California, United States], a high-performance single-board computer designed for embedded applications. The Jetson Nano offers a variety of sensor interfaces, including two MIPI CSI camera connectors, one HDMI 2.0 display port, four USB 3.0 ports, and one Gigabit Ethernet port. The microcomputer also features a general-purpose input/output (GPIO) bus with two $I^{2}C$ interfaces and two SPI interfaces. The extensive set of ports enables the connection of all the peripheral sensors required and simplifies the whole system into a standalone system. The Jetson Nano’s CPU and GPU chips are powerful enough to employ algorithms based on deep learning toolboxes such as DeepLabCut-Live!, developed by Kane et al.^13^ For our initial development efforts, its CPU performance has been sufficient. However, future studies and developments will capitalize on its powerful GPU capabilities.

To visualize the animals’ behavior and physiology in real-time, we employed two miniaturized Raspberry Pi camera modules with IMX219 camera sensor and threaded mount for M12 lenses (B0152, ArduCam, Nanjing, China). To acquire images in the absence of visible light, we specifically utilized camera models without the optional infrared filters. The body camera employs an M12 wide-angle lens with an effective focal length of 1.8 mm and a 100 deg field of view (LS-1820, Arducam) to capture the frontal view of the mouse at a distance of 15 cm. It monitors the animal’s body movements during measurements enabling visual assessment of the animal’s grooming behavior and potential signs of distress [Fig. 1(b)iii]. We utilized the second camera input to monitor pupil diameter as a readout of neural activity and alertness level.^7^^,^^14^^–^^16^ To image the mouse pupil, a telephoto M12 lens with a 21.8-mm effective focal length (CIL121, CommonLands, San Diego, California, United States) was employed, limiting the field of view to 18 deg from a distance of 8 cm [Fig. 1(b)iv]. The IMX219’s 3280×2464 imaging sensor can be programmatically configured to bin pixels and/or crop the field of view to acquire images with a variety of image sizes and either 16:9 or 4:3 aspect ratios. For our device, both cameras were configured to acquire images with a size of 320×240  pixels, enabling memory-efficient frame collection at manufacturer-reported rates of up to 120  frames/s for each camera.

To simplify camera positioning during experimental setup, both cameras are mounted onto ½-inch optical posts held by magnetic universal post holders (Thorlabs, Newton, New Jersey, United States) using custom 3D-printed mounts. A custom light-emitting diode (LED) printed circuit board (PCB) was designed to attach directly to the camera PCB and provide 940-nm infrared illumination for use with experiments sensitive to visible light. In the absence of visible light, mice tend to fully dilate their pupils, thus preventing us from observing fluctuations in pupil size. Consequently, we also provided additional options for visible light illumination (470- and 520-nm LEDs) on the LED board to mildly constrict the pupil [Fig. 1(b)iv].

#### 2.1.3 Signal communication and synchronization between systems

Precise synchronization is required to rigorously analyze and correlate behavioral measurements from our device with brain imaging measurements. A 16-bit analog-to-digital converter chip (ADC, ADS1115, Texas Instruments, Dallas, Texas, United States) was connected to the microcomputer via an $I^{2}C$ interface to record trigger signals and ensure precise timing [Fig. 1(b)v]. The ADC offers four single-end input channels or two differential channels. For our application, one differential channel represents the trigger signal to initiate stimulation trials, whereas the other differential channel records the trigger for individual functional stimuli. As detailed in Sec. 4.2, the functional somatosensory stimuli for the current study consisted of a train of 0.3-s pneumatic air puffs (3 Hz, 3 s total duration) for whisker deflection.

### 2.2 Software Design

#### 2.2.1 GUI on microcomputer

**Fig. 2:**
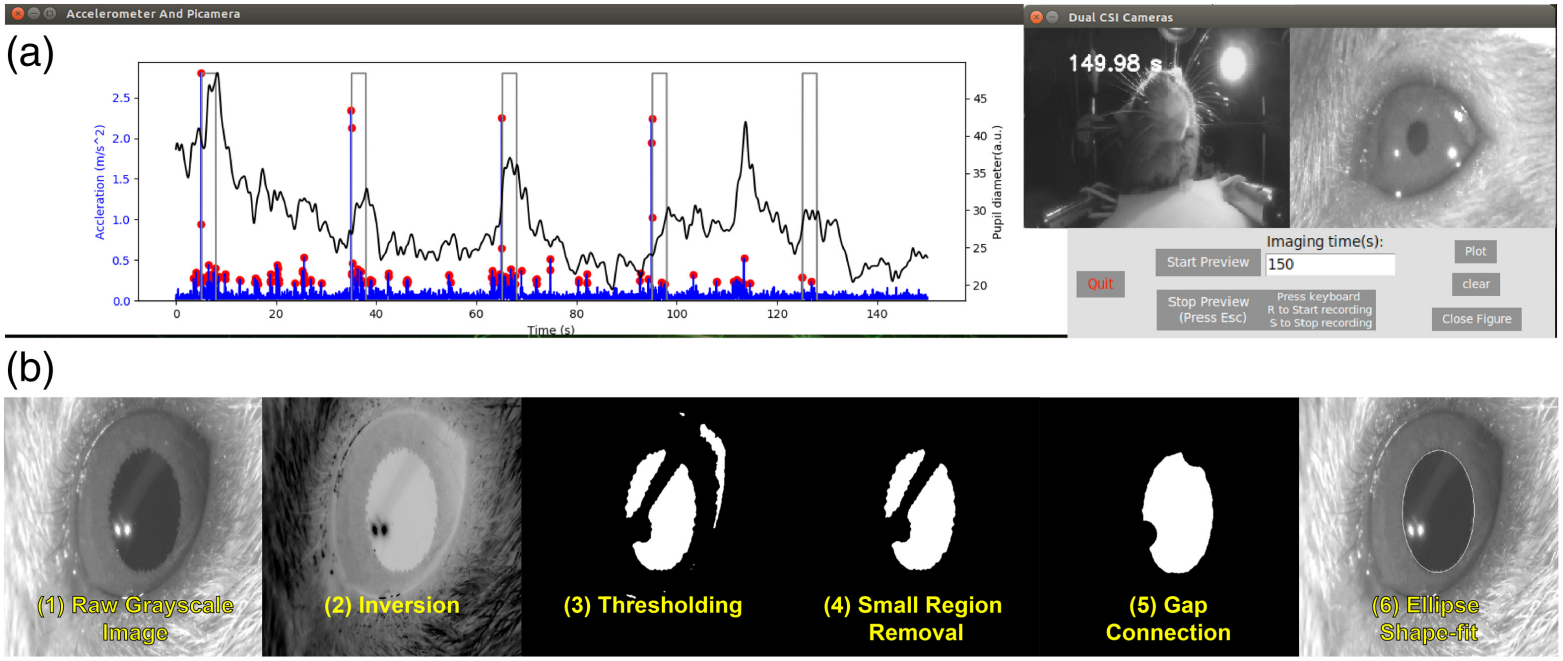
(a) Custom GUI with real-time display of acceleration and frame-wise feedback from two cameras. (b) Stepwise images of the pupillometry algorithm.

A custom graphical user interface (GUI) was developed in Python 3.6.9 with the Tkinter toolbox, specifically for the Jetson Nano microcomputer. The GUI incorporates several features, including (1) real-time display from body and pupil cameras, (2) automatic data acquisition initiated by a 5 V trigger signal, and (3) real-time display of acceleration during data acquisition [Fig. 2(a)]. Immediately after each experimental run, the camera and accelerometer data can be easily reviewed to quickly screen for spontaneous behaviors.

#### 2.2.2 Pupillometry analysis

To monitor dynamic changes in pupil size, we embedded an algorithm developed with the OpenCV library in our custom data acquisition software.^10^ The analysis procedure is developed in Python 3.6.9, and the step-by-step procedure is illustrated in Fig. 2(b). Briefly, after acquiring the raw grayscale image, the pixel value is inverted. Thresholding is then applied to binarize the image. After thresholding, small areas and regions with low circularity are removed to isolate the pupil region. In select frames, momentary whisker twitching will result in the pupil being blocked and inadvertently segmented into two parts. To correct this, the divided pupil region is reconnected and fitted as an ellipse. The pupil diameter is calculated by the number of pixels spanning the minor axis of the ellipse. Pupil diameter is low-pass filtered below 1 Hz with a fourth-order Butterworth filter.

### 2.3 Testing and Validation Via Functional Activation Experiments

#### 2.3.1 Rapid screening of motion artifacts

**Fig. 3:**
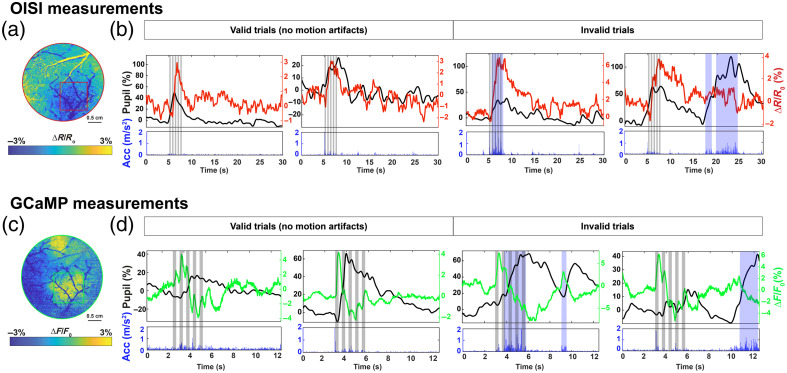
Representative OIS ($\Delta R/R_{0}$) and GCaMP ($\Delta F/F_{0}$) measurements performed using WFOI and our integrated device. (a) OIS imaging of the functional hyperemia center (red rectangle) in the barrel cortex through a 3-mm cranial window. (b) Four representative trials showing 30-s time courses of total hemoglobin change, pupil diameter, and acceleration. (c) GCaMP imaging of the neural activity center (green rectangle) through the same cranial window. (d) Four representative trials showing 12.5-s time courses of GCaMP fluorescence, pupil diameter, and acceleration. Gray shaded bars represent the pneumatic whisker stimulus, and blue indicates spontaneous motion artifacts. The total blood dynamic change signal (red curves) was deliberately inverted to accurately reflect the increase in blood volume.

We tested our integrated device’s utility to quickly detect motion artifacts during functional stimulation experiments. The somatosensory cortices of awake Thy-1 GCaMP6f mice were imaged with our WFOI setup while the integrated device monitored their behavior and measured pupil diameter. Imaging sessions were performed to monitor stimulus-induced changes (pneumatic whisker deflection, 300-ms airpuff, 3-Hz stimulus train, 3-s duration per trial) in blood volume by optical intrinsic signal (OIS) (reflectance measurements λ=568±10  nm, 5  frames/s, 30 s per trial). In separate imaging sessions, stimulus-induced changes in neuronal calcium dynamics were monitored by GCaMP fluorescence (fluorescence measurements $\lambda_{\mathrm{ex}}=470\pm 40\;\mathrm{nm}$, $\lambda_{\mathrm{em}}=525\pm 50\;\mathrm{nm}$, 20  frames/s, 12.5 s per trial). To identify spontaneous motion artifacts, the acceleration recordings were screened in 0.5 s intervals. A motion artifact was defined as any instance during which the acceleration reading exceeded $0.2\;m/s^{2}$ for 200 ms or longer. Figure 3 displays representative results from individual imaging trials with and without motion artifacts. Pneumatic whisker deflection provoked dynamic, local increases in both hemodynamics and neural activity [Figs. 3(a) and 3(b)], consistent with prior studies.^17^^,^^18^ A concomitant increase in pupil diameter was observed for each stimulation trial. Example trials of both viable and corrupted OIS measurements lasting 30 s are displayed in Fig. 3(b), along with GCaMP fluorescence measurements lasting 12.5-s durations, collected separately [Fig. 3(d)]. For several stimulation trials, the whisker stimulus evoked a confounding motion artifact, yielding distinct fluctuations in the accelerometer recordings. Example motion artifacts are highlighted by shaded blue regions in Figs. 3(b) and 3(d). In trials with no motion artifacts, response profiles of cerebral blood volume (CBV, calculated as normalized changes in reflectance, $\Delta R/R_{0}$), neuronal GCaMP fluorescence ($\Delta F/F_{0}$), and pupil diameter resembled well-established measured profiles reported previously.^3^^,^^12^ When spontaneous motion artifacts occurred, unpredictable fluctuations were observed in all readouts. Pupil dilations also persisted for a longer duration in the presence of motion artifacts, reflecting the initiation of the animal’s fight-or-flight response.^19^

#### 2.3.2 Correlating pupillary response with cortical blood volume and neuronal calcium

**Fig. 4:**
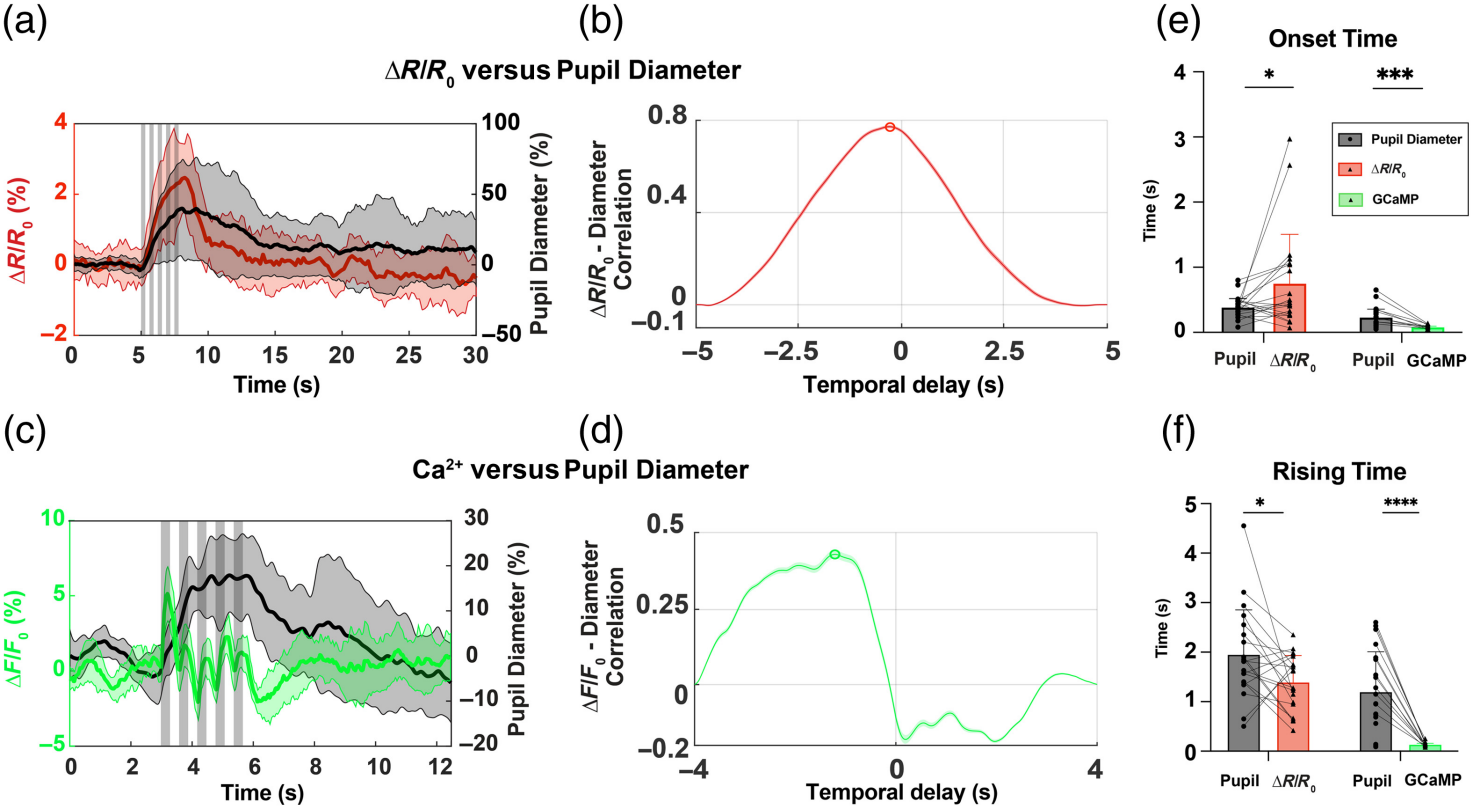
Trial-averaged results of (a) OIS ($\Delta R/R_{0}$) versus pupil diameter and (c) GCaMP ($\Delta F/F_{0}$) versus pupil diameter in response to whisker stimulation. Gray shaded bars indicate stimulus pulses. (b) Cross-correlation between changes in total hemodynamic changes and pupil diameter 5 s after stimulus onset. The red dot indicates the peak correlation value. (d) Cross-correlation between GCaMP fluorescence and pupil diameter 4 s after stimulus onset. The green dot indicates the maximum correlation location. Panel (e) and (f) are the onset time (*p=0.0439 and ***p=0.0003) and rising time (*p=0.0268 and ****p<0.0001) comparisons between pupil diameter and optical results. Statistical difference was tested using Student’s t-test (mean ± SD).

We further validated our device’s performance by analyzing and correlating kinetic features of functional imaging data collected with WFOI and our integrated device. Specifically, we correlated stimulus-induced pupillometry changes acquired from our device with vascular and neuronal measurements from the cortex acquired with WFOI. The data were first screened to remove trials corrupted by spontaneous motion artifacts. Block-averaged time courses of OIS versus pupil diameter and GCaMP versus pupil diameter are displayed in Figs. 4(a) and 4(c). We calculated the cross-correlation between these combinations of signals as well as the onset time and rise time of each signal from Figs. 4(b) and 4(d). The pupil diameter measurement was detrended before cross-correlation. The rising time was defined as the time from 10% to 90% of the peak height after the beginning of the first stimulus, and the onset time was defined as the beginning of the first stimulus to 10% of the peak height.

Figure 4(b) relates the kinetics of stimulus-induced pupillary dilation and cortical blood volume. The responses of cortical blood volume and pupil dilation were strongly correlated (0.77±0.0033). Our onset time analysis indicates that time pupil dilation initiates nearly 300 ms before the change in cortical blood volume [pupil onset time: 0.394±0.168  s, $\Delta R/R_{0}$ onset time: 0.763±0.722  s, Fig. 4(e)]. Pupil diameter and OIS rose from 10% to 90% of their peaks in 1.957±0.928  s and 1.397±0.558  s, respectively [Fig. 4(f)]. The results indicate that, although pupillary dilation initiates earlier than vascular changes in the cortex, pupillary dilation takes longer than cortical blood volume to reach its stimulus-induced peak, in agreement with previous reports.^10^^,^^14^^,^^20^^–^^22^

Our measured pupil diameter changes also correlated well with evoked increases in calcium in cortical Thy1 neurons, with a correlation value of 0.43±0.014 [Fig. 4(d)]. However, the kinetic features of GCaMP fluorescence differed considerably from pupil dilation. Neuronal calcium increased and decreased rapidly in response to each stimulus pulse, whereas the pupil dilated more gradually as the stimulus train persisted. Our calculations indicate that the onset time of neuronal GCaMP fluorescence (0.09±0.03  s) occurred earlier than that of the pupil [0.283±0.15  s, Fig. 4(e)]. The rise time for GCaMP (0.133±0.043  s) was also considerably shorter than pupillary dilation [1.475±0.699  s, Fig. 4(f)].

Taken together, our observations suggest notable differences between the kinetics of stimulus-evoked neuronal and vascular responses in the rodent cortex compared with its pupil diameter. Stimulated changes to pupil diameter appear to initiate earlier than changes in cortical blood volume but not neuronal calcium. Pupil dilation also requires longer to reach its respective stimulus-induced peak than cortical blood volume or neural activity.

## 3 Discussion and Conclusion

Methods and tools for awake animal imaging have grown increasingly necessary to gain detailed insight into the intricate processes that dynamically regulate brain function.^1^^,^^2^ These techniques are especially vital to investigations exploring neurovascular coupling. As a result, recognition has grown for the need to account for spontaneous perturbations to brain activity. Behavioral monitoring is an integral component of virtually all brain imaging experiments in awake subjects, and numerous approaches have been implemented to identify factors that could modulate cerebral hemodynamics and neural activity.^12^^,^^23^ Our custom device easily and effectively permits real-time screening of naturally occurring, intermittent phenomena in small animals that modulate brain activity, such as spontaneous locomotion, whisking, and grooming activity, and variations in alertness. The device consists of inexpensive (<$500), portable components and streamlines measurement and analysis using a powerful microcomputer. The compact sensors can be easily configured and positioned, significantly reducing experimental setup time to under 5 min. The customized, triggerable, user-friendly software provides immediate assessment of behavioral data following acquisition and permits easy, precise synchronization with two-photon microscopes, or other intravital brain monitoring systems. Our pilot results demonstrate that the device can easily identify spontaneous motion artifacts and fluctuations in alertness during functional activation trials. In practice, during an imaging session, the software allows investigators to review behavioral readouts immediately following a sequence of imaging trials. Such rapid feedback affords opportunities to immediately determine whether more stimulus trials can or should be acquired during each experiment session, potentially improving the throughput and success rate of awake imaging studies.

We coupled our device with WFOI and precisely correlated pupil dilation dynamics with cortical blood volume dynamics and Thy1 neuronal activity. Monitoring pupil diameter has been extensively utilized in humans and animals as a robust readout of neuronal activity patterns governing alertness and arousal.^14^^,^^24^^,^^25^ We observed that the onset of stimulus-evoked pupil dilation lags the associated onset of cortical neuronal activity by ∼190  ms. Our findings agree well with established reports that measured pupil diameter and cortical neuron activity during fluctuations in alertness or spontaneous treadmill activity. The delay between cortical neural activation and pupil response reflects the complex parasympathetic and sympathetic pathways that modulate smooth muscle constriction and relaxation in the eye.^16^^,^^26^ Conversely, our observations showed that stimulus-evoked pupil dilation leads to a corresponding increase in cortical blood volume by ∼300  ms. Similarly, Turner et al.^10^ observed that spontaneous pupil dilation preceded fluctuations in CBV by ∼1  s in alert mice and mice during the REM and NREM stages of sleep. We hypothesize that smooth muscle signaling and vascular transit time largely account for the delayed CBV response relative to pupil dilation.^27^

Irrespective of onset time, we found that stimulus-evoked pupillary dilation requires more time to reach its peak than either cortical blood volume or neural activity. Prior studies demonstrated that increased activity from both adrenergic and cholinergic neurons precedes pupillary dilation,^16^ whereas cortical astrocytic activity follows pupillary dilation.^28^ These and other findings illustrate the intricate balance of precisely timed signaling between multiple cell types in different neuromodulatory systems that contribute to regulating alertness and arousal state. Although extensive investigations are required to rigorously characterize the influence of multiple neuromodulatory circuits on spontaneous or stimulus-evoked cellular and hemodynamic activity in the brain,^19^^,^^29^ our device shows promise as a simple and affordable instrument to facilitate these comprehensive studies.

One limitation of our validation experiments comes from not accounting for the influence of the partial blood volume effect [Fig. 4(b)].^30^ For each stimulation trial, the stimulus-induced increase in blood volume reduced the peak magnitudes of neuronal GCaMP fluorescence signal (from ∼5% to ∼1%) for later stimulus pulses. However, the partial volume effect’s impact on the calcium signal’s onset and rise time was minimal and likely did not appreciably affect our kinetic analysis. Multiple methods exist to robustly account for the reduction in fluorescence signal by hemoglobin’s absorption.^31^^,^^32^ Our current findings motivate more comprehensive investigations relating neuronal, vascular, and pupil kinetics using multispectral OISI/fluorescence imaging. In future experiments, partial volume correction will be implemented on all measurements by regressing the fluorescent measurement with 530-nm reflectance measurements.^31^^,^^32^

Our device shows promise for extensive and easy monitoring of dynamic behavioral events in animals during awake imaging sessions. Future developments will focus on extending the device’s capabilities to increase the sampling rate and measure more peripheral physiological sensors. Specifically, to enable robust tracking of the animal’s whisker and to potentially monitor respiration and heart rate, technical improvements will focus on improving both cameras’ optical resolution and frame rates. Prior reports emphasized the strong influences of a stimulated whisker’s initial position, angle of deflection, and neighboring whisker activity on the resultant cortical response.^33^^–^^35^ To explore this further, the body camera’s magnification and frame rate will be increased up to 100 to 150 fps. To investigate the potential, yet disputable, relationship between pupil fluctuations and respiration rate,^36^^,^^37^ pupil camera’s frame rate and magnification will also be increased to 100 fps or higher. Additional efforts will explore modifications to utilize our device during MRI and PET experiments. Future developments will also extend our software’s capabilities to operate with more affordable and widely utilized micro-computers.

## 4 Methods

### 4.1 Animal Preparation

All experiments were performed in accordance with ARRIVE guidelines for animal care, under a protocol approved by the Northeastern University Institutional Animal Care and Use Committee (Protocol # 22-1239R). Female and male Thy1-GCaMP6f mice are used in this study (n=5, 3 months old, weight = 20 to 30 g, Jackson Laboratory, Strain #:025393, Bar Harbor, Maine).

Each mouse underwent craniotomy following previous procedures.^6^^,^^38^ To reduce inflammation and edema during and after surgery, dexamethasone (4.8  mg/kg at 4  mg/ml) and cefazolin (0.5  g/kg at 200  mg/ml) were administered 4 h before surgery. The mouse was anesthetized with isoflurane (3% to 4% for induction and 1% to 2% for maintenance) during the surgery. The mouse head was fixed and secured to the stereotactic frame (David Kopf Instruments, Tujunga, California, United States), and the heating pad (Harvard Apparatus, Holliston, Massachusetts, United States) was placed under the mouse to maintain its body temperature. Mouse’s hair and scalp were removed for surgery preparation. A 3-mm diameter cranial hole was made in the left somatosensory barrel cortex using a dental drill. After exposing the dura, a custom acrylic coverslip plug was inserted to cover the intact brain tissue and was sealed with Loctite 401. A custom head post was secured on the right skull with Loctite 401. The entire skull area was then covered with dental cement. During post-surgical recovery, the mouse was single-housed, supported with antibiotics (40/8  mg/ml, sulfamethoxazole/trimethoprim (SMX-TMP) and 50  mg/ml, carprofen in drinking water), and administered cefazolin (0.5  g/kg at 200  mg/ml) continuously for 5 days. Mouse head-fixed habituation and whisker stimulation training was carried out for progressively longer durations for 7 days after the recovery. By incrementally increasing the training time from 5 to 45 min, the mice gradually acclimatized to receiving whisker stimulus while their heads were immobilized.

### 4.2 Functional Activation Experiment

For sustained deflection of the whisker, a pneumatic drug injection system (PDES-DXH, ALA Scientific, Farmingdale, New York, United States) was used to deliver air puffs (20 psi) through a narrow glass tube. A custom 3D-printed nozzle was utilized to disperse the air and deflect a greater proportion of the whisker without inducing eye blinking. Each OIS imaging trial consisted of 5 s of pre-stimulation, 3 s of stimulation, and 22 s of post-stimulation (30 s in total). Similarly, GCaMP imaging trials consisted of 3 s of pre-stimulation, 3 s of stimulation, and 6.5 s of post-stimulation (12.5 s in total). Whisker stimulation was conducted with a pulse duration of 300 ms and an interval of 300 ms (five stimulations).

### 4.3 Imaging Setup

The experiments were performed with a customized WFOI system (Fig. S1 in the Supplementary Material). Using different optical filter sets, the system is capable of measuring either OIS-reflecting variations in total hemoglobin or GCaMP fluorescence signals. A 568±10  nm light source was used to illuminate the exposed cortex for collecting the relative change in hemoglobin concentration. Similarly, 470±40  nm light was used to excite the GCaMP calcium indicator. The reflected photons were collected by a 4× objective (NA=0.20, WD=20  mm, Nikon, Tokyo, Japan) and detected by a CMOS camera (acA 1300−200  μm, Basler, Exton, Pennsylvania, United States). An emission filter (525/50  nm) was used to limit the excited photons only for calcium fluorescence imaging. OIS and calcium fluorescence data were required at 5 and 20 fps with 20- and 45-ms exposure time, respectively.

### 4.4 Workflow Analysis

Programmed electronic outputs are preset for synchronization among the different systems. When sending pulses from the control panel, the integrated device, the pneumatic machine, and the optical imaging camera begin operating simultaneously. The device collects frames from both cameras and records acceleration and the triggers for whisker stimulus pulses. The imaging camera is triggered at the beginning of each repeated trial and records the reflected/excited signal from the cranial window. In each trial, the pneumatic machine is set to gently deflect the mouse whisker periodically, resulting in functional activation on the barrel cortex. The flowchart is shown in Fig. S2 in the Supplementary Material.

### 4.5 Statistical Analysis

The number of measurement trials in Sec. 2.3.2 is n=20 in OISI measurement and n=16 in GCaMP calcium imaging from animals prepared in Sec. 4.1. Cross-correlation was used to analyze the temporal relationship and correlation coefficient between optical signals and pupil diameter during stimulation using function “*xcorr*” in MATLAB R2024a (MathWorks, Natick, Massachusetts, United States). Two-tailed, independent student’s t-test (mean ± SD) was performed to identify significant differences between optical signals and pupil diameter on the onset and rising time using Prism version 10 (GraphPad, Boston, Massachusetts, United States; *P<0.05, ***P<0.001, and ****P<0.0001).

## Supplementary Material



## Data Availability

Commercial components and custom software developed for the study can be found at https://github.com/orgs/yaseen-OMNIlab/. Additional details can be provided upon request.
