# Supplementary material for: Low-cost physiology and behavioral monitor for intravital imaging in small mammals
Source: Neurophotonics. 2025 Jan 25;12(1):015004. doi: 10.1117/1.NPh.12.1.015004 (PMC11759666; doi:10.1117/1.NPh.12.1.015004)
Supplement: Supplementary file 1 [file NPh_012_015004_SD001.pdf]

Supplementary material:

Table 1 Integrated platform components

Part Name	Manufacturer	Part Number	Quantity	Unit Cost
4GB Jetson Nano Developer kit B01	NVIDIA, USA	945-13450-0000-100	1	$230
Raspberry Pi Camera V2	Arducam, China	B0152	2	$35
Telephoto M12 lens	CommonLands, USA	CIL121	1	$59
ADC	Adafruit, USA	ADS1115	1	$15
PCB fabrication	PCBWay, China			$100
**Total Cost**				$439

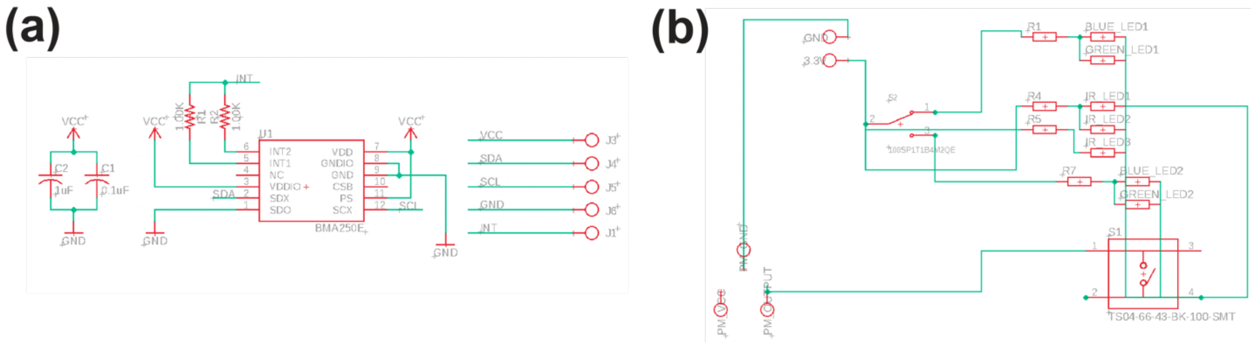

Fig. 1 Accelerometer board (a) and LED sensor (b) electronic circuit's schematics.

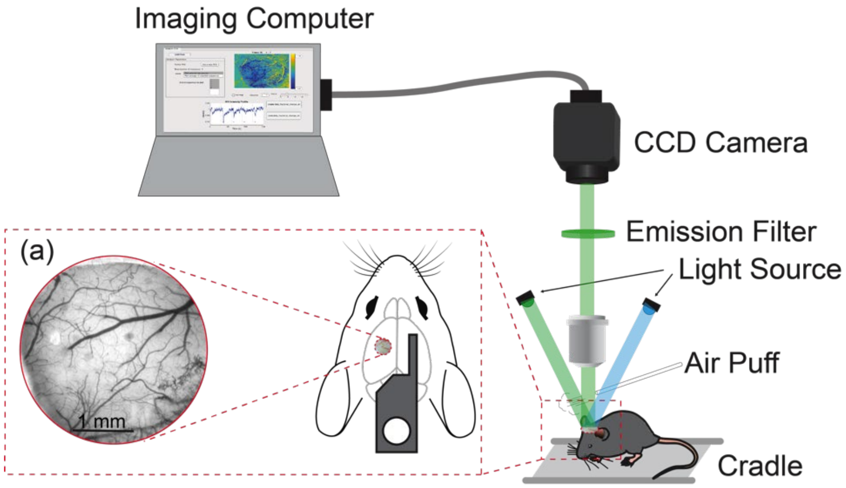

Fig. 2 Schematic of the WFOI for OIS and GCaMP flourenscence calcium imaging. The reflected/excited signals are detected by a 4X objective and collected by CMOS camera. The emission filter is utilized when imaging the flourenscence signal. The pneumatic machine delivers air puffs to periodically deflect mouse right whiskers. (a) A representative cranial window on the mouse somatosensory barrel cortex. Scale bar: 1 mm.

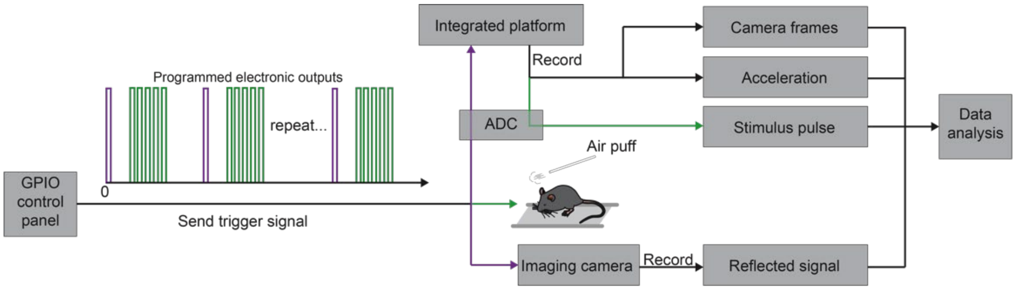

Fig. 3 Workflow of synchronizing systems and data. The purple pulse is programmed to trigger the integrated platform and the optical imaging camera, and the green pulse is periodically sent to the pneumatic machine to deliver air puff to the whisker and also recorded by the integrated platform.
